# In Vitro Protein Digestion of Cooked Spent Commercial Laying Hen and Commercial Broilers Breast Meat

**DOI:** 10.3390/foods11131853

**Published:** 2022-06-23

**Authors:** Thanatorn Trithavisup, Pornnicha Sanpinit, Sakhiran Sakulwech, Annop Klamchuen, Yuwares Malila

**Affiliations:** 1 National Center for Genetic Engineering and Biotechnology (BIOTEC), Thailand Science Park, Tambon Khlong Nung 12120, Thailand; kamonrat.tri@ncr.nstda.or.th (T.T.); pornnicha.san@ncr.nstda.or.th (P.S.); 2 National Nanotechnology Center (NANOTEC), Thailand Science Park, Tambon Khlong Nung 12120, Thailand; sakhiran.sak@nanotec.or.th (S.S.); annop@nanotec.or.th (A.K.)

**Keywords:** in vitro digestion, protein digestibility, GC-MS, particle size, commercial broiler, spent laying hen

## Abstract

Chicken meat from spent laying hens (SHs) has been considered as nutritive as the meat of commercial broilers (CBs) based on chemical composition. High insoluble collagen in SH meat might reduce protein digestibility and bio-accessibility compared to CB meat. This study aimed at comparing the in vitro protein digestibility of CB and SH cooked breast meat. In the first part, CB samples were digested using two static in vitro digestion methods and collected at different digestion points for determining the degree of hydrolysis (DH). The method providing a greater DH value was chosen for comparing protein digestibility between CB and SH samples. The activities of used enzymes during in vitro digestion were evaluated based on bicinchoninic acid assay 2,4,6-trinitrobenzenesulfonic acid colorimetric method, gas chromatography-mass spectrometry, and sodium dodecyl sulfate-polyacrylamide electrophoresis. Particle size distribution of solid content collected from hydrolysate was also determined. The results showed that after digestion, CB showed 1–3 mg/mL protein concentration lower, while 7–13% DH and 50–96 µmoL/g protein-free NH2 groups higher when compared to those of SH. Based on sodium dodecyl sulfate-polyacrylamide electrophoresis, CB samples exhibited greater intensity of band at MW < 15 kDa than that of SH. Regarding particle size in terms of volume weighted mean (D[4,3]), at the end of the oral phase, the end of the gastric phase, and the beginning of the intestinal phase, D[4,3] of the SH samples were 133.17 ± 2.16, 46.52 ± 2.20, and 112.96 ± 3.63 µm, respectively, which were greater than those of CB (53.28 ± 1.23, 35.59 ± 1.19, and 51.68 ± 1.25 µm). However, at the end of the intestinal phase, D[4,3] of SH and CB, which were 17.19 ± 1.69 and 17.52 ± 2.46 µm, respectively, did not significantly differ from each other. The findings suggested a greater in vitro protein digestibility of cooked CB breast meats than that of SH ones.

## 1. Introduction

The United Nations (UN) recently released population projections of 8.5 billion by 2030, 9.7 billion by 2050, and exceeding 11 billion by 2100 [1]. The increase in the world population may lead to food shortages, especially food protein. Currently, many studies have focused on the use of alternative proteins [2,3,4,5]. However, the use of food waste or by-products from the food industry would be another option for meeting the future nutritional needs of a growing global population.

Chicken breast meat is widely recognized as an inexpensive protein source and contains important essential amino acids and organic nitrogen [6,7]. Spent laying hens (SHs) are a massive by-product from poultry and egg industries [8,9] mostly used in pet food and not much for human consumption [10,11]. This is due mainly to its undesirable tough texture when the meat is cooked [12]. The toughness of SH meat has been shown to be associated with high total and heat-stable collagen content, densely-packed small muscle fibers, and a lower degree of myofibril fragmentation [12].

Lakshani et al. [8] compared the chemical composition of the meat from SHs and commercial broilers (CBs) and reported higher protein and lower moisture in minced SH breast compared to those of CB samples. No significant differences in crude fat and ash contents were detected between commercial broilers and the spent hens. Total polyunsaturated fatty acid content was significantly higher in spent hen meat compared to that of broiler meat, in particular eicosapentaenoic acid and docosahexaenoic acid. Based on the findings, Lakshani et al. [8] concluded that the nutritional value of SH and CB meat was comparable; therefore, SH meat would be another inexpensive source of food protein.

However, the chemical concentration is not the sole determinant of nutritional the quality of food. In addition, the ingested protein quality depends greatly on their composition and concentration in essential amino acids and the capacity of an organism to absorb amino acids and peptides during the digestion process [13]. In this regard, a high insoluble collagen content might affect the digestibility and bio-accessibility of SH meat. However, detailed studies on the digestibility properties of the SH meat are still limited.

Digestibility was one of the key parameters defining the quality of a given dietary protein. Several methods or models, including static and dynamic in vitro models, various cell and ex vivo cultures, animals and humans have been used for an assessment of protein digestibility. Although in vivo methods for protein digestibility combine the assessment of digestibility, bio-accessibility and bioavailability, in vivo methods are expensive, time-consuming, labor intensive, and entail ethical problems. Therefore, an in vitro digestion method mimicking gastrointestinal behavior is widely used as an alternative to in vivo experiments. The in vitro digestion method simulated gastrointestinal conditions in the human system including oral, gastric, and duodenal digestion. For protein digestion, a large part of protein digestion starts in the stomach in which pepsin plays an important role in protein digestion. Pepsin catalyzes the breakdowns of intact protein into peptides with four to nine amino acids [14]. In the small intestine, the combined action of proteases (i.e., trypsin, elastase, carboxypeptidase, and chymotrypsin) hydrolyze peptides into smaller peptides and free amino acids. The tripeptides, dipeptides, and amino acids are absorbed into the bloodstream through the small intestine [14].

In 2014, the COST infogest network outlined a static INFOGEST in vitro digestion method [15] with an attempt to set the conditions to closely resemble the physiological situation and harmonize the practical protocols used among studies. However, they underlined that the proposed conditions might not be suitable for all research questions and should be validated for specific application. Egger et al. [16] indicated that the INFOGEST model required modification of some steps to enable quantification and comparison of protein digestibility in different laboratories and different food matrices. In 2014, Bordoni et al. [17] studied the foodomics approach for the evaluation of protein bio-accessibility in processed meat upon in vitro digestion. The in vitro method described in Bordoni et al. [17] was similar to the conditions and processes that occur in vivo, and considers three main phases: oral, gastric, and duodenal digestion. They suggested that this protocol can be applied to study the effects of digestion and compare the different digestibility and bioavailability of protein-rich foods. Hence, the in vitro digestion method described by Bordoni et al. [17] was selected for comparing the protein digestibility of chicken breast meat to INFOGEST protocol.

The objectives of this study were to compare the in vitro protein digestibility of cooked SH and CB breast meat. Prior to the comparison, the protein digestibility of cooked CB breast meat was conducted with two available in vitro enzymatic digestion procedures to determine the suitability of the methods. The protein digestibility of breast meats was evaluated through bicinchoninic acid (BCA) assay and sodium dodecyl sulfate polyacrylamide gel electrophoresis (SDS-PAGE) assays, trinitrobezenesulfonic (TNBS) acid method, gas chromatography-mass spectrometry (GC-MS) technique, and particle size determination.

## 2. Materials and Methods

### 2.1. Materials

Breast meat from commercial broilers (CBs) and spent commercial laying hens (SHs) were provided by a local slaughter house (Nakhon Ratchasima, Thailand). The meat was frozen and kept at −18 °C while being transported to the Food Biotechnology laboratory, BIOTEC (Pathum Thani, Thailand). Upon arrival, the meat was stored at −18 °C until further analyses. The samples were thawed at 4 °C overnight. Subsequently, the breast samples were individually packed in a plastic bag and cooked at 95 °C in water bath until temperature of the thickest part of the sample reached 75 °C [12]. The samples were then cooled in iced water until the temperature was reduced to 15 °C and placed for at least 1 h at 4 °C. The cooked samples were then minced for 2 min using a blender and stored at −30 °C until further analyses. Prior to any experiment, the samples were thawed overnight at 4 °C.

The moisture of the cooked chicken breasts was determined using a halogen moisture analyzer (HX204, Mettler-Toledo, Switzerland). The crude protein of the cooked meat was examined following the Kjeldahl method [18]. A factor of 6.25 was used in a calculation for crude protein. The moisture and crude protein content of the cooked CB were 69.15% and 28.82%, respectively, whereas the cooked SH contained 64.79% moisture content and 37.78% crude protein.

Chemicals and solvents were analytical grade and purchased from Carlo Erba Re-agenti (Rodano, Italy), Merck (Darmstadt, Germany), and Sigma-Aldrich (St. Louis, MO, USA). The α-amylase (EC: 3.2.1.1, 100,000 U/g) was purchased from Megazyme (Wick-low, Ireland). Pepsin (EC: 3.4.23.1, ≥3200 Units/mg), pancreatin (EC: 232–468-9, 4 × USP activity), and bile salt were purchased from Sigma-Aldrich (St. Louis, MO, USA).

### 2.2. Experimental Design

This experiment was divided into two parts. The first part aimed at comparing the protein digestibility of cooked CB meat (*n* = 4) using either an INFOGEST [15] or the method described by Bordoni et al. [17], labeled herein as M1 and M2, respectively. Each in vitro enzymatic reaction was carried out in duplicate.

In the second part, the in vitro protein digestibility of cooked SH and CB breast meat was compared. A total of 8 samples (4 SH breasts, 4 CB breasts) were subjected to an in vitro enzymatic hydrolysis following the method of Bordoni et al. [17]. An in vitro enzymatic reaction was carried out in duplicate.

### 2.3. In Vitro Enzymatic Digestion

For INFOGEST (M1), simulated salivary fluid (SSF), simulated gastric fluid (SGF), and simulated intestinal fluid (SIF) solutions were prepared with a composition as shown in Table 1. The pH of the SSF, SGF, and SIF solutions was adjusted to pH 7.0, pH 3.0, and pH 7.0, respectively, using 6N HCl and 1N NaOH. To simulate oral digestion, 5 g of minced cooked CB samples were mixed with 3.5 mL of SSF electrolyte solution and 75 U/mL α-amylase for 2 min in a 50 mL centrifuge tube. During all steps of the enzymatic digestion, the samples were stirred in a trayster digital (IKA^®^ TRAYSTER digital, IKA Works, Inc., Staufen, Germany) at 10 rpm and kept at 37 °C. Then, 7.5 mL of SGF electrolyte solution containing pepsin 2000 U/mL was added. The pH of the mixture was then adjusted to pH 3.0 by adding 6N HCl. After 120 min incubation at 37 °C, 11 mL of SIF electrolyte solution with pancreatin (100 U/mL final concentration based on trypsin) and bile salt (10 mM final concentration) was added, and the pH of the mixture was increased to pH 7.0 by adding 1 M NaOH. The digestion was proceeded for 240 min. During the digestion procedure, the samples (2 mL) were collected at the end of oral digestion (M1-P1), the end of the gastric digestion (M1-P2), after 120 min of intestinal digestion (M1-P3), and after 240 min of intestinal digestion (M1-P4). The enzymatic reaction of each collected sample was stopped by incubating the samples in boiling water for 2 min. The samples were then centrifuged at 3000× *g* for 30 min, and the supernatant was filtered through 0.2 mm membranes. All samples were stored at −20 °C for further analyses.

As for the second procedure (M2) described by Bordoni et al. [17], an enzymatic reaction was performed inside a 50 mL centrifuge tube, stirred by trayster digital (IKA^®^ TRAYSTER digital, IKA Works, Inc., Staufen, Germany) at 10 rpm and kept at 37 °C as conducted for INFOGEST. A buffer solution (pH 6.9), containing 120 mM NaCl, 5 mM KCl, and 6 mM CaCl_2_ was added in proper volumes at every step. To simulate oral di-gestion, CB samples (2 g dry basis) were mixed with 4 mL of buffer solution containing 75 U/mL α-amylase. After 5 min amylase digestion, 8 mL of the buffer solution was added, and the pH of the mixture was then decreased to pH 2.0 by adding 6N HCl. Gastric digestion was started by adding pepsin (a final concentration of 2000 U/mL) to the sample. After 60 min incubation at 37 °C, 8 mL of the buffer solution was added, and the pH was increased to pH 5.0 with 1.5 M NaHCO_3_ to stop pepsin digestion. Intestinal digestion started with the addition of pancreatin (100 U/mL final concentration based on trypsin) and bile salt (10 mM final concentration) to the mixture. The pH was adjusted to pH 6.0 with 1.5 M NaHCO_3_, and digestion was followed for 180 min and 300 min. During the digestion procedure, 2 mL of the mixture was collected as follows; M2-P1 at the beginning of gastric digestion (after decreasing pH to 2.0, and before adding pepsin); M2-P2 at the end of the gastric digestion; M2-P3 at the beginning of the intestinal phase (after the increase of pH to 5.0, before the addition of pancreatin and bile salt); M2-P4 after 180 min of intestinal digestion; and M2-P5 after 300 min of intestinal digestion. To stop enzymatic reaction, 35% NaOH was added to the samples collected at M2-P1 and M2-P2 until final pH was increased to pH 8.0. The samples collected at M2-P3, M2-P4, and M2-P5 were acidified to pH 2.0 with 6N HCl to stop pancreatic hydrolysis. All samples were centrifuged at 3000× *g* for 30 min, and the supernatant was filtered with 0.2 mm membranes. The supernatants and sediments of all samples were stored at −20 °C until further analyses.

**Figure 1:**
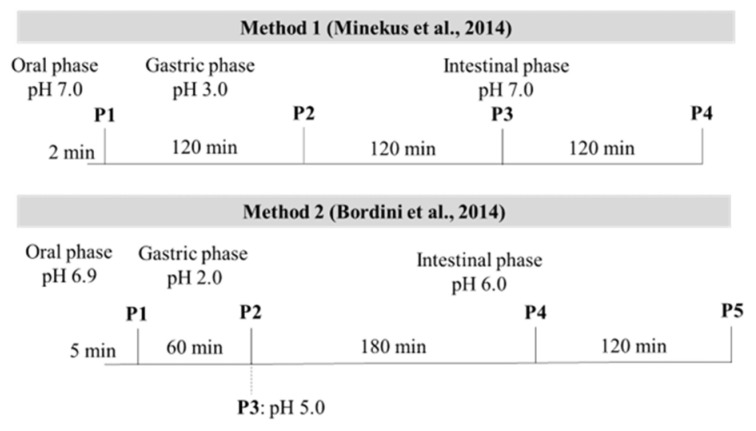
pH and digestion time of in vitro method 1 [15] and 2 [17] at end of oral phase (M1-P1 and M2-P1), end of gastric phase (M1-P2 and M2-P2), and before (M2-P3), half (M1-P3 and M2-P4), and end of intestinal phase (M1-P4 and M2-P5).

The different pH and digestion time at each digestion point of both in vitro methods are shown in Figure 1.

### 2.4. Bicinchoninic Acid (BCA) Assay Quantitative Analysis

The concentration of protein in the supernatants collected from each digestion point was determined using a Pierce^TM^ BCA Protein Assay Kit (Thermo Fishes Scientific, Rockford, IL, USA) according to the manufacturer’s instruction and as previously described [19]. Bovine serum albumin (BSA) at different concentrations were used as a protein standard. All supernatants from in vitro digestion as well as standards were performed in 96 well plates (in duplicates). The 10 µL of sample or standard solutions were added to each well followed by 200 µL of reagent A and B (reagent A:B = 25:1). The sealed plate was wrapped in aluminum foil and incubated at 37 °C for 30 min. The absorbance at 562 nm was read in a microplate reader (SpectraMax Plus 384, Molecular Devices, LLC, Sunnyvale, CA, USA).

### 2.5. Trinitrobezenesulfonic (TNBS) Acid Method

Free NH_2_ groups and degree of protein hydrolysis (%DH), defined as the proportion of cleaved peptide bonds, were examined using a TNBS method as previously described [20]. Supernatants from in vitro digestion were diluted 1:200 with 1% sodium dodecyl sulfate (SDS). Leucine solutions of 0, 0.075, 0.15, 0.3, 0.6, 0.9, 1.2, and 1.5 mM in 1% SDS were used for standard curve. The assay was performed in 96-well plates and 15 µL of samples or standard solutions (in duplicate) were added to each well followed by 45 µL 0.21 M sodium phosphate buffer (pH 8.2) and 45 µL TNBS solution (0.05% *w*/*v* in water). The sealed plate was wrapped in aluminum foil and incubated at 50 °C for 1 h. The reaction was stopped by the addition of 90 µL 0.1N HCl and absorbance at 340 nm was read in a microplate reader (SpectraMax Plus 384, Molecular Devices, LLC, Sunnyvale, CA, USA). Free NH_2_ content was expressed in the unit of micromoles per gram protein, as leucine amino equivalents, after subtraction of a blank. The degree of protein hydrolysis (%DH) was calculated using an equation as follows.

%DH = h/h_tot_ ×100% (1)

h = (leucine NH_2_ − β)/α (2)

where α = 1.00, β = 0.40 [21], h_tot_ = 7.6 mmoL/g protein [22].

### 2.6. Gas Chromatography-Mass Spectrometry (GC-MS)

Profiles of free amino acids in the supernatant was examined using a GC-MS in accordance with the method described by Jiménez-Martín et al. [23] with modification. The sample (400 mg), in duplicates, was mixed with 4 mL of 25% acetonitrile in 0.1N HCl and sonicated at room temperature for 20 min. After centrifugation at 9000 rpm for 20 min, 150 µL of supernatant was transferred into a GC vial. Norleucine (50 µL), used as an in-ternal standard, was then added into the vial. The solution was dried using a concentrator (Concentrator plus, Eppendorf, Germany) for 2 h and derivatized with 50 µL of N-tert-butyldimethylsilyl-N-methyltrifluoroacetamide containing 1% tert-butyldimethyl chlorosilane (Sigma-Aldrich) and 50 µL acetonitrile at 100 °C for 4 h. The derivatized samples were subsequently analyzed using a GC 7890A/MS 5975C System (Agilent Technologies, Palo Alto, CA, USA). One microliter of the derivatized sample was injected in spitless mode onto a 50 m × 0.25 mm i.d., 0.1 µm, DB-5 column (Agilent Technologies, Palo Alto, CA, USA). Injector and MS transfer line temperatures were set at 280 °C and 300 °C, respectively. Carrier gas was helium at a flow rate of 1.2 mL/min. Oven temperature was maintained at 170 °C for 5 min initially, raised to 200 °C at 4 °C/min, then held isothermal for 3 min and finally raised to 285 °C at 4 °C/min. Ion source temperature was 230 °C and ionization energy was 70 eV. Scanning was performed using a mass range from 35 to 800 m/z. The standard mixture of amino acids with known concentrations were prepared to generate calibration curves where peak areas of each amino acid were plotted against the known concentrations for each amino acid in the standard mixture. The amino acid content was calculated and expressed as milli-gram per 100 g sample.

### 2.7. Sodium Dodecyl Sulfate Polyacrylamide Gel Electrophoresis (SDS-PAGE)

Electrophoretic patterns of the digested protein were determined using SDS-PAGE as previously described [24]. The supernatants at different digestion points equivalent to 20 µg protein were mixed with 2 µL of 5× sample buffer, containing 5% SDS, 50% glycerol, 0.1% bromophenol blue, 250 mM Tris-HCl, pH 6.8 and 5% betamercaptoethanol, and then boiled at 95 °C for 5 min in a water bath. The mixtures were cooled to room temperature and 10 µL of the protein mixture were loaded on to a 4–20% Mini-PROTEAN TGX stain-free gels (Bio-Rad Laboratories, Richmond, CA, USA). Precision Plus Protein^TM^ Unstained Protein Standards with molecular weight 10–250 kDa (Bio-Rad Laboratories, Richmond, CA, USA) was loaded (5 µL) alongside the samples as a molecular weight marker. Electrophoresis was carried out for 75 min at constant 120 V. The gel was subsequently activated under a UV trans illumination mode a Gel Doc™ XR+ imaging system. The image of protein bands was acquired using an Image Lab™ software version 6.0.1 (Bio-Rad Laboratories).

### 2.8. Particle Size Distribution

Particle size of the sediment was analyzed using a Malvern™ Mastersizer 2000 instrument (Malvern Instruments Ltd., Worcestershire, UK). The cooked CB and SH breasts and sediment from different points were diluted in distilled water to a final concentration of 1% (*w*/*v*). The volume mean diameter, D[4,3] (µm) was calculated and reported by a Mastersizer 2000 software (Malvern Instruments Ltd., Worcestershire, UK).

### 2.9. Statistical Analysis

To compare the effects of different in vitro methods, the mean values within each digestion point were compared using Student’s *t*-test. Differences in in vitro protein digestibility at each digestion point between CB and SH were assessed using Student’s *t*-test in similar manner. To analyze the difference in protein concentration, free NH_2_ content, %DH and free amino acid content among different digestion points within each method or each chicken type, one-way analysis of variance (ANOVA) was performed. Mean differences were then analyzed using Duncan’s new multiple range test. All statistical analyses were performed by IBM SPSS statistics software (Windows, SPSS Inc., Chicago, IL, USA). The significance level was set at *p* < 0.05.

## 3. Results and Discussion

### 3.1. Effects of Different In Vitro Digestion Methods on Protein Digestibility of Cooked CB Breast Meat

**Figure 2:**
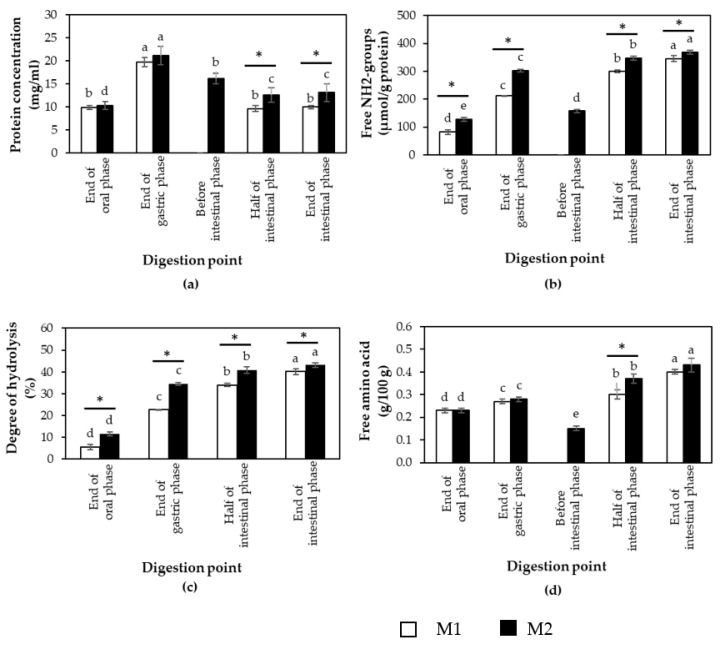
(**a**) Protein concentration; (**b**) free NH_2_-groups; (**c**) degree of hydrolysis (%); (**d**) free amino acid contents of commercial broiler breasts digested by method 1 (M1) and method 2 (M2) and collected at end of oral phase, end of gastric phase, and before, half, and end of intestinal phase. Bars and error bars represent mean and standard deviation (*n* = 4), respectively. Asterisks above bars indicate a significant difference between the in vitro methods at particular phase (*p* < 0.05). Different letters indicate significant difference among different digestion points (*p* < 0.05).

Meat protein is usually denatured by heat in a temperature range of 50–85 °C [25]. Thermal denaturation of protein can also induce structural changes leading to an increase in protein surface hydrophobicity [26,27], a loss of protein solubility, and an increase in protein–protein aggregation [28]. According to BCA assay (Figure 2a), the data showed that at the intestinal phase, protein concentration of CB digested using M1 was significantly lower than that of CB digested using M2 (*p* < 0.05). The results suggested that protein in the CB sample was hydrolyzed within M2 at a greater extent compared to M1.

Considering protein concentration at each phase (Figure 2a), protein concentration at oral phase (P1) in the supernatants of both M1 and M2 was approximately 10 mg/mL. At this phase, the minced cooked CB breasts were mixed with α-amylase which mimicked the enzymatic action in a human mouth. The α-amylase hydrolyzed α-glycosidic linkage in the starch molecule but did not hydrolyze the peptide bond. Therefore, the protein contents in this phase might be from protein solubilization of cooked CB in the digestion buffer.

At the gastric phase (P2), the results showed that the protein concentration in digested samples from both M1 and M2 was twice as high compared to those of the samples in the oral phase (Figure 2a). Moreover, this point had the highest protein concentration among all digestion points. The result was agreed with Bordoni et al. [17]. They found that after Bresaola, an Italian dried salted meat, was digested by M2 in this study, the greatest protein concentration, determined based on Bradford assay, of Bresaola was observed at P2. The key enzyme of this phase is pepsin, an endopeptidase which randomly hydrolyzes peptide bonds within protein molecules to produce relatively large peptides when compared to oligopeptides and free amino acid in the small intestine phase. Bordoni et al. [17] addressed that during pepsin digestion, and two parallel phenomena occurred: (1) protein solubilization from the bolus mass led to increasing total content of detectable proteins, and (2) a part of proteins was hydrolyzed to peptides having MW < 5 kDa and became not detectable. In this study, the protein concentration of the hydrolyzed samples was determined with a BCA assay. The increased protein concentration at P2 suggested that the solubilization of proteins from the bolus mass was the predominant phenomenon.

M2-P3 was the point that prepared before the intestinal phase. The pH of digested solutions was adjusted closer to *pI* [17]. The protein concentration of the samples at this point decreased when compared to M2-P2 (Figure 2a). The results might be due to the effects of pH adjustment.

M1-P3, M1-P4, M2-P4, and M2-P5 mimicked the intestinal phase. The protein concentration (Figure 2a) of the CB digested samples significantly decreased when compared to their counterparts in the gastric phase (*p* < 0.05). In this phase, buffer solution, pancreatin, and bile salt were added to the solution. Pancreatin, the main enzyme in this phase, consisted of trypsin, ribonuclease, and other proteases. After this phase, the protein or peptide in the samples were hydrolyzed and became oligopeptides, tripeptides, dipeptides, and free amino acids [14]. Therefore, the reduction of protein concentration is related with the low molecular weight fraction of digested sample due to enzymatic hydrolysis.

The appearance of free NH_2_ groups was another technique used as a measure of protein digestibility. The generation of free NH_2_ groups through the cleavage of peptide bonds can be estimated by employing TNBS (trinitrobenzensulfonic acid) as a reactive reagent [29]. The result from Figure 2b showed that at all digestion phases, free NH_2_ groups of CB digested by M1 were significantly lower than those of M2 (*p* < 0.05). At the gastric phase (P2), although the digestion time of M1 was longer than the digestion time of M2 (Figure 1), the free NH_2_ groups of M1 was still lower than that of M2. The results suggested that CB samples in M2 underwent hydrolysis at a greater extent than that in M1. At the gastric phase of M1, the buffer system was not stable. The pH of the digested solution from M1 increased from 3.0 to 3.5 by 30 min, and reached a pH of approximately 5.0 by 120 min. Therefore, the digested solutions had to adjust the pH to 3.0 every 30 min. The main enzyme in this phase was pepsin which was mainly active between pH 2 and 4 [15]. Minekus et al. [15] addressed that in the gastric phase; the pH value must be checked and adjusted throughout the digestion process or use pH stat because the buffer is not suitable for all situations. This might disrupt the digestion process, thus resulting in lower protein digestibility.

The changes in free NH_2_ groups during the digestion of the CB samples in M1 and M2 exhibited a similar trend. The lowest free NH_2_ groups were found at the oral phase and the free NH_2_ groups had significant increases in every digestion point (*p* < 0.05).

As for %DH (Figure 2c), the results showed that at all digestion points, CB digested by M1 showed significantly lower %DH compared to CB digested by M2 (*p* < 0.05). The %DH of samples digested by both M1 and M2 (Figure 2c) increased in every digestion point (*p* < 0.05).

In this study, free amino acids released during in vitro digestion were identified using GC-MS and the summation of their quantity are presented in Figure 2d. The results indicated that only at the intestinal phase, total free amino acid content of CB digested by M1 had significantly lower than that of CB digested by M2 (*p* < 0.05). However, the total free amino acid content of samples from both M1 and M2 exhibited profiles similar to the results of free NH_2_ groups. The increase in total free amino acid content after in vitro digestion was generated by digestive hydrolysis.

**Figure 3:**
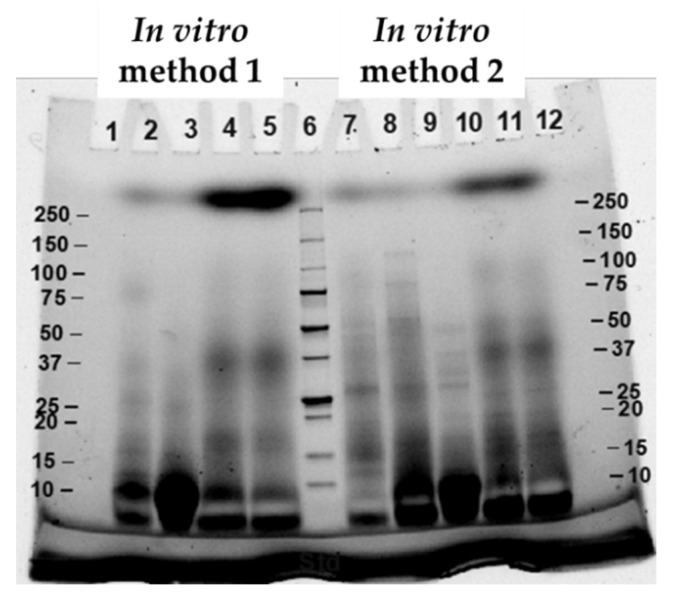
SDS-PAGE of commercial broiler breasts (CB) digested by method 1 (lane 2–5) and method 2 (lane 7–11) and collected at end of oral phase (lane 2 and 7), end of gastric phase (lane 3 and 8), and before (lane 9), half (lane 4 and 10), and end (lane 5 and 11) of intestinal phase. Lane 6 was protein maker.

Patterns of digested proteins at different digestion points from both M1and M2 were examined using SDS-PAGE (Figure 3). The results showed that at M1-P1 (lane 2) and M2-P1 (lane 7), broad molecular weight (MW), ranging from <10 to 75 kDa for M1 (lane 2) and from <10 to 150 kDa for M2 (lane 7) were observed. After pepsin digestion or at the end of gastric phase (P2), the bands with the most intensity from both digestion methods were at MW < 20 kDa (lane 3 for M1-P2 and lane 8 for M2-P2). As for at the end of intestinal phase (lane 4–5 and 10–11), the most intense bands were at <10 kDa. The results indicated that protein in cooked CB samples were hydrolyzed to a greater extent when they were subjected to each step of the digestion method. This SDS-PAGE profile corresponded well with the results of free NH_2_ group, %DH, and free amino acids. The different MW profiles from the two in vitro methods might be due to protein solubility in different digestion buffer solutions.

**Figure 4:**
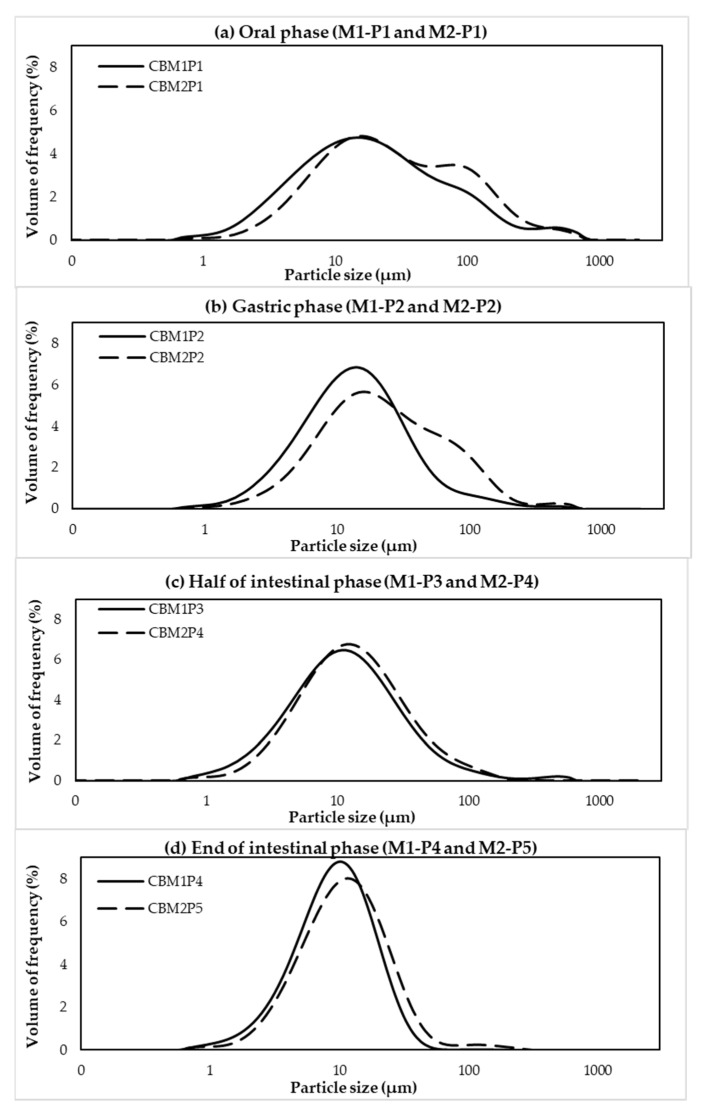
Particle size distribution of commercial broiler breasts (CB) digested by method 1 (M1) and method 2 (M2) and collected at end of oral phase (**a**); end of gastric phase (**b**); and half (**c**); and end (**d**) of intestinal phase.

To observe whether the size of the cooked chicken meat proteins was reduced during the in vitro digestion, size distribution of the pellet collected from the digested samples was determined using a Mastersizer 2000. Overall, the size distribution curves (Figure 4) of both methods were shifted towards the left, indicating reduced particle size of the cooked meat during enzymatic hydrolysis. The results were in agreement with a previous study of Liu et al. [30] in which Sturgeon treated by low temperature vacuum heating and digested by pepsin showed decreased in D[4,3] value. Comparing between the methods, at oral and gastric phases, the D[4,3] values of digested CB by M1 had significant smaller size than digested CB by M2 (*p* < 0.05) but no significant differences were observed at M1-P3, M1-P4, M2-P4 and M2-P5 (*p* > 0.05) (Table 2). However, the particle size distribution of both digestion methods showed the same trend. Before digestion, the CB samples contained three major fractions with D[4,3] of 216.33 µm. After oral (P1, Figure 4a) and gastric digestion (P2, Figure 4b), two or three major fractions with D[4,3] 45.50 µm for M1-P1 and 53.28 µm for M2-P1 and 22.96 µm for M1-P2 and 35.59 µm for M2-P2 were found, respectively. These D[4,3] values were much smaller than those found in the CB sample before digestion. It should be noted that D[4,3] value of M2-P3 (51.68 µm) was greater than the value of M2-P2 (35.59 µm). This might be due to protein aggregation when pH of the solution was adjusted closer to their isoelectric point (*pI*) [17]. The findings were in accordance with decreases in protein concentration (Figure 2a), free NH_2_ content (Figure 2b), and free amino acid content (Figure 2d) observed at M2-P3 in comparison to those of M2-P2. Only one fraction with smaller D[4,3] was found in the CB samples after pancreatin digestion (intestinal phase, Figure 4c,d, M1-P3, M1-P4, M2-P4, and M2-P5).

Overall, the results suggested that M2 is more suitable than M1 for digesting cooked chicken breast. This might be due to the suitable buffering system provided by M2 during the digestion process. In this study, at the gastric phase of M1, the buffer system was not stable leading to readjust pH of the solutions every 30 min. This issue was related with what Minekus et al. [15] described. They stated that after food intake at the gastric phase, the pH usually increased to 5 or above due to the buffering capacity. In the literature, the recommended pH at this phase was pH 3. It was the optimum pH for optimal gastric enzyme activity. Hence, the pH may have to be readjusted during digestion. This is one of limitations of the M1 protocol. In addition, the CB digested by M2 showed higher %DH when compared to CB digested by M1. Therefore, M2 was selected for the next experiment.

### 3.2. Protein Digestibility of Cooked SH and CB Breast Samples during In Vitro Digestion

**Figure 5:**
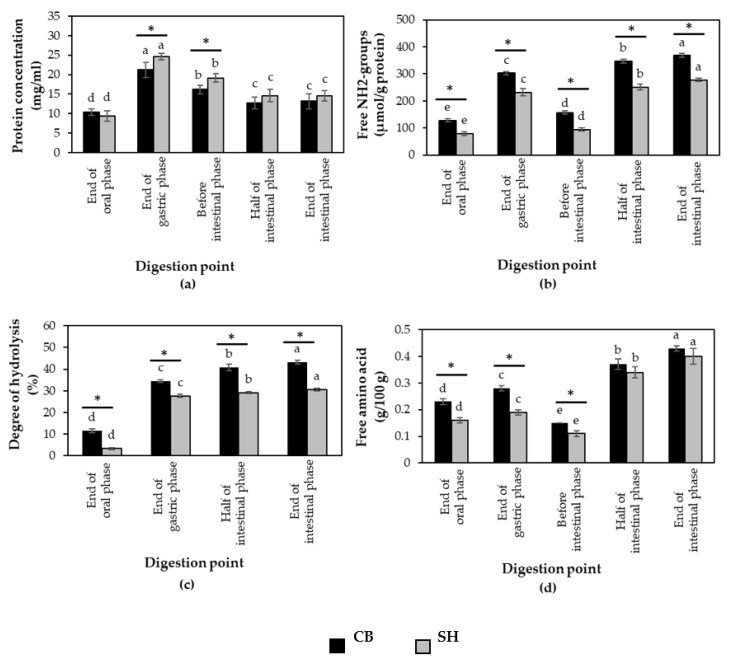
(**a**) Protein concentration; (**b**) free NH_2_-groups; (**c**) degree of hydrolysis (%); (**d**) free amino acid contents of commercial broiler breasts (CB) and spent commercial laying hen breasts (SH) digested by method 2 and collected at the end of oral phase, end of gastric phase and before, half and end of intestinal phase. Bars and error bars represent mean and standard deviation (*n* = 4), respectively. Asterisks above bars indicate significant difference between the chicken types at particular phase (*p* < 0.05). Different letters indicate significant difference among different digestion points (*p* < 0.05).

To compare protein digestibility of cooked SH and CB breast meat, the samples from each chicken breeds were subjected to in vitro digestion method (M2). As shown in Figure 5, both meat types showed similar changes in protein concentration, free NH_2_ groups, % DH and total free amino acid content during the digestion. Although SH showed significantly higher protein concentration than CB at P2 and P3, the samples exhibited significantly lower free NH_2_ groups, %DH and free amino acid content than those of CB at every digestion point (*p* < 0.05). The higher crude protein in cooked SH sample (37.78 g/100 g) compared to cooked CB breast (28.82 g/100 g) might explain the higher values of protein concentration of digested SH samples determined by BCA assay. Moreover, the total content of detectable proteins was increased by protein solubilization as Bordoni et al. [17] described. This might be another reason for the high protein concentration of SH samples. The lower values of free NH_2_ groups in SH samples implied the lower degree of cleaved peptide bonds during in vitro digestion, suggesting that cooked SH sample was enzymatically hydrolyzed at a lesser extent com-pared to cooked CB meat.

In this study, free amino acid profiles of the digested samples at P1 and P5 were com-pared (Table 3). At P1, the predominant free amino acid of both cooked CB and SH was glutamic acid. The findings were in agreement with previous studies addressing that glutamic acid was the predominant essential amino acid in raw broiler and spent hen meats, whereas the predominant non-essential amino acid was alanine [31]. Free amino acids have been involved in the characteristic tastes of food [32]. Glutamic acid is one of the most important amino acids which enhances the umami flavor of chicken meat [33]. Threonine, serine, glutamic acid, glycine, and alanine are related to the taste of food, while valine, leucine, isoleucine, methionine, phenylalanine, proline, and arginine are related to the tangy flavor in meat [34]. The result implied that at P1, cooked CB was higher in flavor and taste compounds than cooked SH.

Focusing on amino acid profiles at P5 (Table 3), among the essential amino acids, threonine, histidine, and tryptophan were significantly lower in cooked SH (*p* < 0.05), whereas non-essential amino acids, alanine, glycine, proline, serine, aspartic acid, hydroxyproline, and glutamic acid was found to be comparatively lower in the cooked SH meat (*p* < 0.05) (Table 3). The results of amino acid composition indicated that cooked CB meat was the better protein source, as it contained higher amounts of some essential amino acids, and overall has superior nutritional meat quality compared to the cooked SH meat.

**Figure 6:**
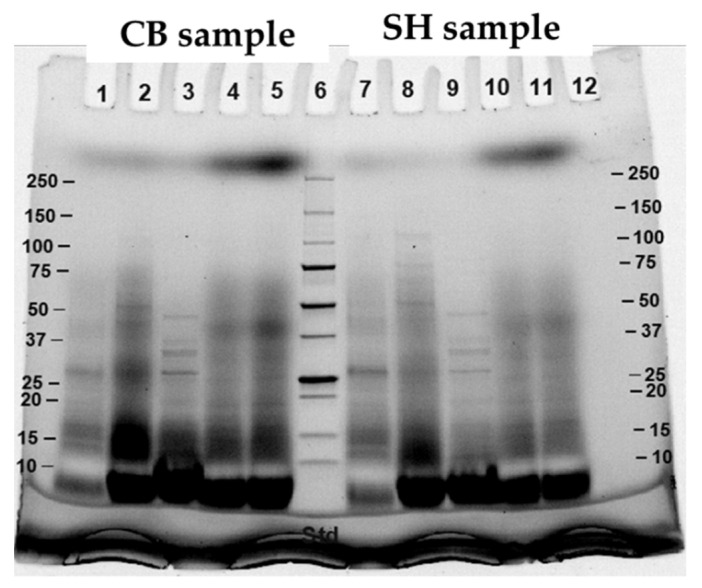
SDS-PAGE of commercial broiler breasts (CB) (lane 1–5) and spent commercial laying hen breasts (SH) (lane 7–11) digested by method 2 and collected at end of oral phase (lane 1 and 7), end of gastric phase (lane 2 and 8), and before (lane 3 and 9), half (lane 4 and 10), and end (lane 5 and 11) of intestinal phase. Lane 6 was protein maker.

SDS-PAGE pattern of digested proteins from cooked SH and CB samples is shown in Figure 6. The results showed that at P1, digested SH samples had greater high-MW fractions when compared to CB samples. After pepsin digestion (P2), SH samples still had band at MW 75–100 kDa and had narrow band at MW 10–20 kDa when compared to CB samples. The results were corresponded to high protein concentration and low free NH_2_ groups, %DH, and free amino acid content of SH sample. However, at the end of intestinal phase (P5), the protein in SH samples still had narrow band of low-MW fraction when compared to protein in CB samples. The result suggested that SH samples were less susceptible to hydrolysis by gastrointestinal enzymes than CB samples.

**Figure 7:**
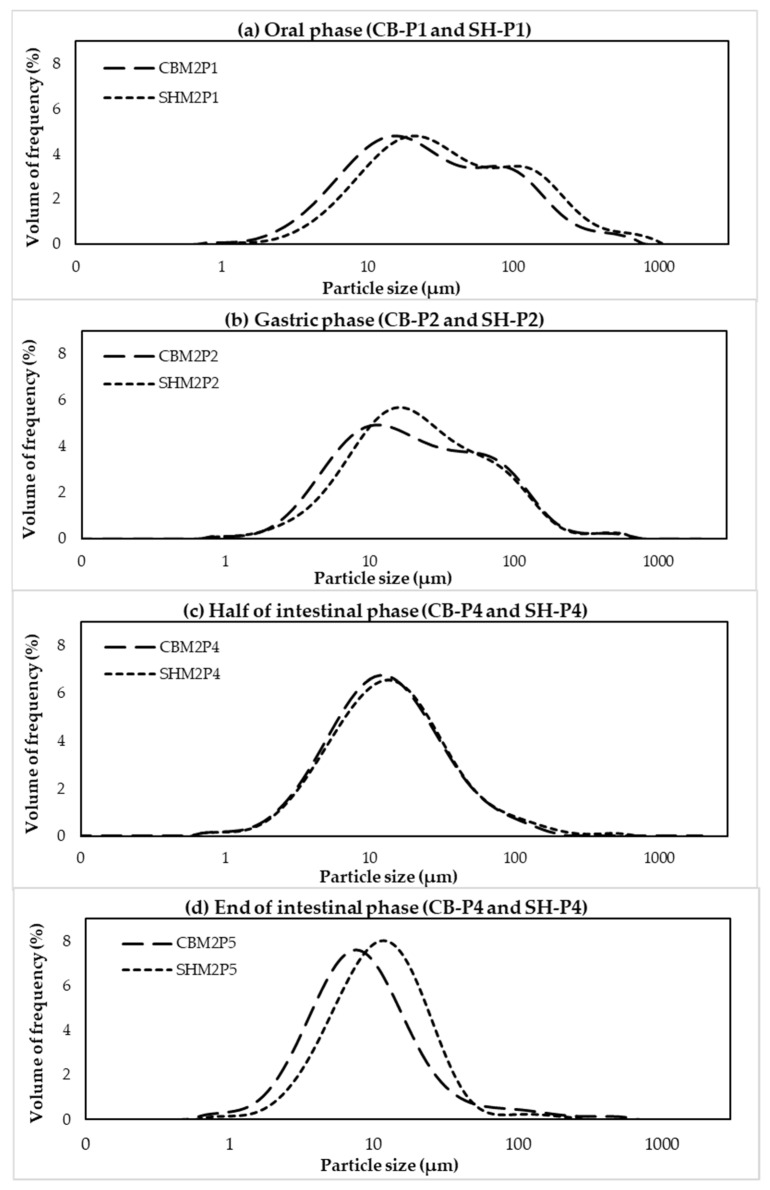
Particle size distribution of commercial broiler breasts (CB) and spent commercial laying hen breasts (SH) digested by method 2 and collected at end of oral phase (**a**), end of gastric phase (**b**), and half (**c**) and end (**d**) of intestinal phase.

Considering particle size distribution (Figure 7), size distribution of both cooked SH and CB samples were shifted towards the lower particle size as the in vitro digestion progressed. However, at oral (P1, Figure 4a) and gastric (P2, Figure 4b) phases, the D[4,3] values of CB were lower than those of SH samples (*p* < 0.05) (Table 4), supporting the lower in vitro protein digestion of cooked SH breasts compared to CB samples.

The lower in vitro protein digestibility of cooked SH samples in comparison to cooked CB samples might be related with the structure and composition of protein within the SH samples. During animal maturation, the collagen fibers stability was increased through covalent cross-links within or between collagen molecules [35]. The divalent cross-links that are usually present in young animals are heat-labile, and they are converted to heat-stable trivalent cross-links as animal ages [36]. Because these trivalent crosslinks are extremely stable [37,38], they are unlikely to be cleaved by most proteinases [39]. It might block enzymatic action, leading to lower hydrolysis reaction of SH sample. To our knowledge, this study is the first to compare the in vitro protein digestibility of cooked SH and CB breasts. Previously, Lakshani et al. [8] reported higher protein content along with polyunsaturated fatty acids in SH raw breast meat compared to those of CB meat and concluded that the SH meat was more nutritious than CB meat. However, our current study suggests that although cooked SH breast contains a greater crude protein content than CB meat, the enzymatic digestion of that protein in cooked SH breast appears to be at a lower extent in comparison to cooked CB breasts. This phenomenon might further reduce the bioavailability of protein from SH meat in vivo.

## 4. Conclusions

In this study, the in vitro protein digestion of cooked chicken breasts was determined using the methods, mimicking the action in the gastrointestinal track, either proposed by INFOGEST (M1) or from Bordoni et al. [17] (M2). The current results indicated that the latter method (M2) is more suitable for determining the protein digestion of cooked chicken meat. The M2 method was then used for a comparison of in vitro protein digestion between cooked SH and CB breasts. The results suggest that cooked SH breast meat might be more resistant to in vitro enzymatic digestion than the CB sample. The changes of chemical bonds of the proteins during digestion is under investigation using a Nuclear Magnetic Resonance (NMR) to obtain more insights into the mechanisms involved.

## Figures and Tables

**Table 1:** Preparation of stock solutions of simulated digestion fluids. The volumes are calculated for a final volume of 500 mL for each simulated fluid.

Constituent	Stock Concentration (mol/L)	SSF (pH 7) (mL)	SGF (pH 3) (mL)	SIF (pH 7) (mL)
KCl	0.5	15.1	6.9	6.8
KH_2_PO_4_	0.5	3.7	0.9	0.8
NaHCO_3_	1.0	6.8	12.5	42.5
NaCl	2.0	-	11.8	9.6
MgCl_2_(H_2_O)_6_	0.15	0.5	0.4	1.1
(NH_4_)_2_CO_3_	0.5	0.06	0.5	-
CaCl_2_(H_2_O)_2_	0.3	0.09	1.3	0.7

**Table 2:** Volume weighted mean (D[4,3]) of commercial broiler breasts (CB) digested by method 1 (M1) and method 2 (M2) 2 and collected at different digestion point.

Digestion Point	D[4,3] (µm) ^1,2^		Effect of Different In Vitro Methods within Each Digestion Point ^3^
	M1	M2	
End of oral phase	45.50 ^a^ ± 0.04	53.28 ^a^ ± 1.23	*
End of gastric phase	22.96 ^b^ ± 0.96	35.59 ^b^ ± 1.19	*
Before intestinal phase	-	51.68 ^a^ ± 1.25	-
Half of intestinal phase	22.03 ^b^ ± 1.75	20.20 ^c^ ± 3.38	ns
End of intestinal phase	19.59 ^b^ ± 2.19	17.19 ^c^ ± 1.69	ns

^1^ The data are presented as mean ± standard deviation. ^2^ Different letters indicate statistically significant difference among different digestion points (*p* < 0.05). ^3^ Asterisks indicate statistically significant difference (*p* < 0.05) between digestion methods, and ns was not significantly different (*p* ≥ 0.05).

**Table 3:** Free amino acid profile from gas chromatography-mass spectrometry (GC-MS) analysis of commercial broiler breasts (CB) and spent commercial laying hen breasts (SH) digested by method 2 and collected at end of oral phase (P1) and end of intestinal phase (P5).

Amino Acid (mg/100 g)	CBP1 ^1^	SHP1 ^1^	Significant Difference ^2^	CBP5 ^1^	SHP5 ^1^	Significant Difference ^2^
**Essential amino acids**						
Valine	6.1 ± 0.5	5.3 ± 1.4	ns	5.2 ± 0.1	4.5 ± 0.5	ns
Leucine	8.6 ± 0.3	7.8 ± 0.8	ns	25.8 ± 0.0	27.0 ± 1.3	ns
Isoleucine	4.7 ± 0.3	3.9 ± 0.2	*	5.4 ± 0.2	5.1 ± 0.3	ns
Methionine	3.7 ± 0.1	3.5 ± 0.5	ns	6.4 ± 0.4	6.6 ± 0.4	ns
Threonine	8.2 ± 0.8	5.5 ± 0.5	*	5.7 ± 0.5	3.7 ± 0.3	*
Phenylalanine	5.8 ± 0.5	5.2 ± 0.6	ns	22.6 ± 4.9	28.7 ± 1.7	ns
Lysine	10.2 ± 0.3	7.2 ± 1.1	*	32.1 ± 2.4	33.0 ± 2.9	ns
Histidine	7.7 ± 0.3	7.1 ± 1.3	ns	5.9 ± 0.2	5.4 ± 0.4	*
Tryptophan	11.4 ± 0.6	9.3 ± 0.3	*	25.8 ± 1.1	19.2 ± 3.2	*
**Non-essential amino acids**						
Alanine	10.8 ± 1.5	6.4 ± 1.2	*	7.2 ± 0.8	4.4 ± 0.4	*
Glycine	8.1 ± 1.1	3.5 ± 0.2	*	11.0 ± 0.6	9.1 ± 0.3	*
Proline	26.9 ± 0.8	16.2 ± 0.8	*	31.3 ± 2.1	26.5 ± 1.3	*
Serine	7.3 ± 0.7	4.8 ± 0.9	*	4.8 ± 0.5	3.1 ± 0.3	*
Aspartic acid	6.7 ± 1.0	3.5 ± 1.1	*	4.0 ± 0.7	2.0 ± 0.4	*
Hydroxyproline	1.1 ± 0.1	0.5 ± 0.0	*	0.8 ± 0.1	0.4 ± 0.0	*
Cysteine	2.4 ± 0.1	2.2 ± 0.1	*	3.2 ± 0.2	3.0 ± 0.2	ns
Glutamic acid	32.6 ± 2.4	21.5 ± 5.9	*	15.0 ± 1.4	10.7 ± 2.3	*
Arginine	3.2 ± 0.5	1.9 ± 0.5	*	11.4 ± 0.9	10.6 ± 0.6	ns
Tyrosine	10.3 ± 0.3	8.4 ± 0.9	*	37.3 ± 1.9	37.9 ± 2.4	ns

^1^ The data are presented as mean ± standard deviation. ^2^ For the effect of different chicken types within each digestion point; * indicate statistically significant difference (*p* < 0.05), and ns was not significantly different (*p* ≥ 0.05).

**Table 4:** Volume weighted mean (D[4,3]) of commercial broiler breasts (CB) and spent commercial laying hen breasts (SH) digested by method 2 and collected at different digestion point.

Digestion Point	D[4,3] (µm) ^1,2^		Effect of Different Chicken Types within Each Digestion Point ^3^
	CB	SH	
End of oral phase	53.28 ^a^ ± 1.23	133.17 ^a^ ± 2.16	*
End of gastric phase	35.59 ^b^ ± 1.19	46.52 ^c^ ± 2.20	*
Before intestinal phase	51.68 ^a^ ± 1.25	112.96 ^b^ ± 3.63	*
Half of intestinal phase	20.20 ^c^ ± 3.38	22.45 ^d^ ± 2.74	ns
End of intestinal phase	17.19 ^c^ ± 1.69	17.52 ^d^ ± 2.46	ns

^1^ The data are presented as mean ± standard deviation. ^2^ Different letters indicate statistically significant difference among different digestion points (*p* < 0.05). ^3^ Asterisks indicate statistically significant difference (*p* < 0.05) between chicken types and ns was not significantly different (*p* ≥ 0.05).

## Data Availability

The data presented in this study are available on request from the corresponding author.
